# Supplementary material for: FAM3D is essential for colon homeostasis and host defense against inflammation associated carcinogenesis
Source: Nat Commun. 2020 Nov 20;11:5912. doi: 10.1038/s41467-020-19691-z (PMC7679402; doi:10.1038/s41467-020-19691-z)
Supplement: Supplementary file 1 — Supplementary Information [file 41467_2020_19691_MOESM1_ESM.pdf]

Supplementary Information

FAM3D Is Essential for Colon Homeostasis and Host Defense against Inflammation Associated Carcinogenesis

Liang et al.

Supplementary Figure 1

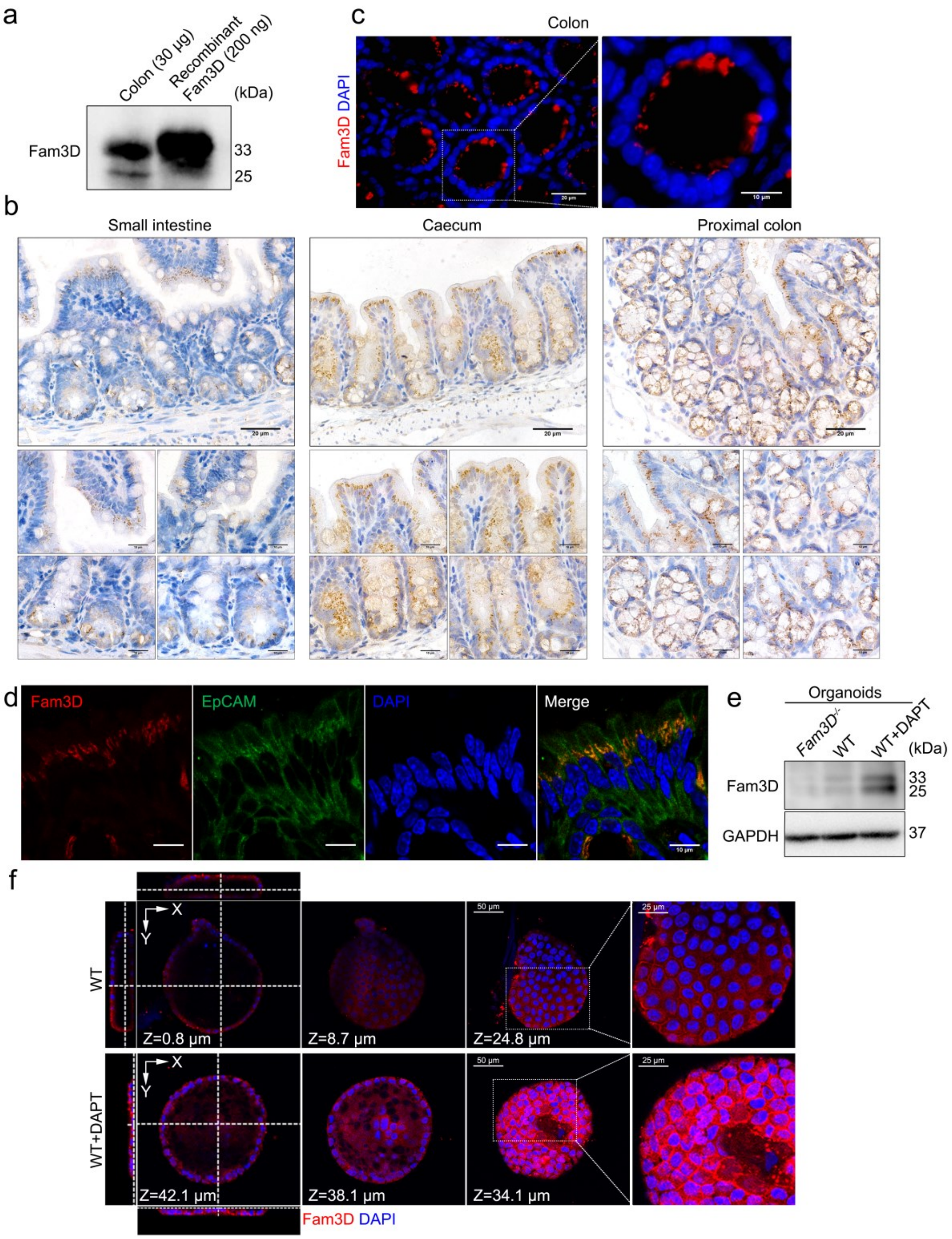

Supplementary Figure 1. Constitutive expression of Fam3D in mouse gastrointestinal tract

a. Quantification of relative Fam3D level in mouse colon tissues measured by Western blot. **b.** Representative immunohistochemical staining for Fam3D in mouse small intestine and cecum. Brown color indicates Fam3D. Scale bar = 20 µm; Insert scale bar = 10 µm. **c.** Immunofluorescent staining for Fam3D (red) in mouse colon. Scale bar = 20 µm; inset scale bar = 10 µm. **d.** Dual immunofluorescent staining for Fam3D (red) and EpCAM (green) in WT mouse colon. Scale bar = 10 µm. **e**. Western blot of Fam3D in organoids treated with or without the Notch inhibitor DAPT. **f.** Immunofluorescence of Fam3D in WT mouse colon organoids in the absence (upper panels) or presence (lower panels) of DAPT. Data is representative of 1 experiment repeated 3 independent times.

Supplementary Figure 2

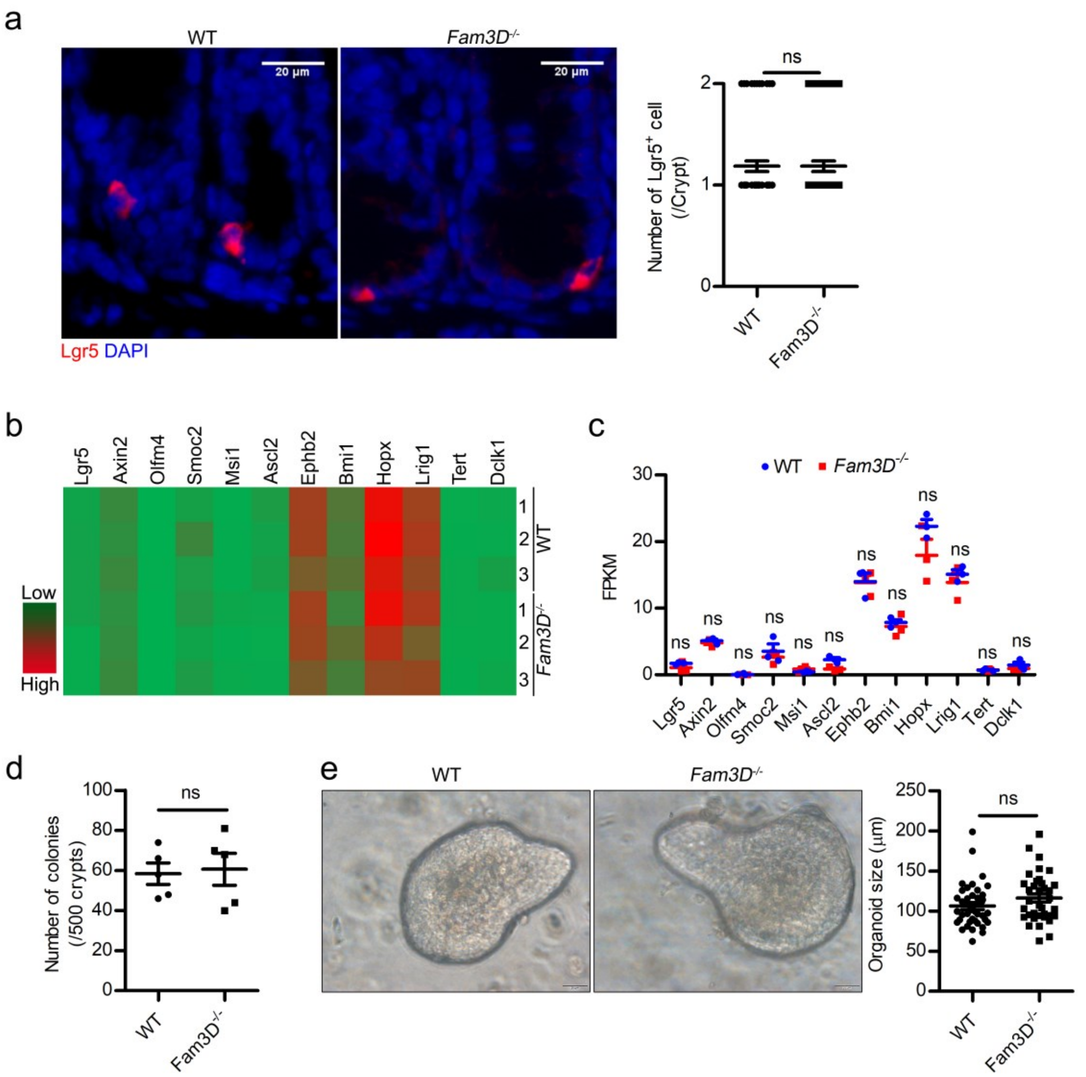

Supplementary Figure 2. No effect of Fam3D on the renewal of colonic mucosal layer

a. Representative immunohistochemical staining for Lgr5 (red) in mouse colon. Scale bar = 20 μm. Distal sections of colons obtained from 4 WT or *Fam3D*$^{-/-}$ mice. Lgr5 staining showing the length of colonic crypts (11 to 20 crypts from each colon). **b.** Heatmap of stem cell related genes in the colon of WT and *Fam3D*$^{-/-}$ mice. **c.** FPKM of stem cell related genes in the colon of WT and *Fam3D*$^{-/-}$ mice. Genes differentially expressed were indicated (> 1 $\log_2$ FC and < -1 $\log_2$ FC, < 0.05 adjusted *P* value), ns, no significance. *n* = 3 for each group. FPKM: Fragments Per Kilobase Million. **d.** The formation of organoids from isolated crypts of WT and *Fam3D*$^{-/-}$ mice. *n* = 5 for each group. **e.** The size of organoids from isolated crypts of WT and *Fam3D*$^{-/-}$ mice at day 7 in culture. Data are representative of 1 experiment examined over 2 independent experiments. Data is presented as the mean ± SEM. Statistical significance was determined by unpaired, 2-tailed Student's *t* test, ns, no significance.

Supplementary Figure 3

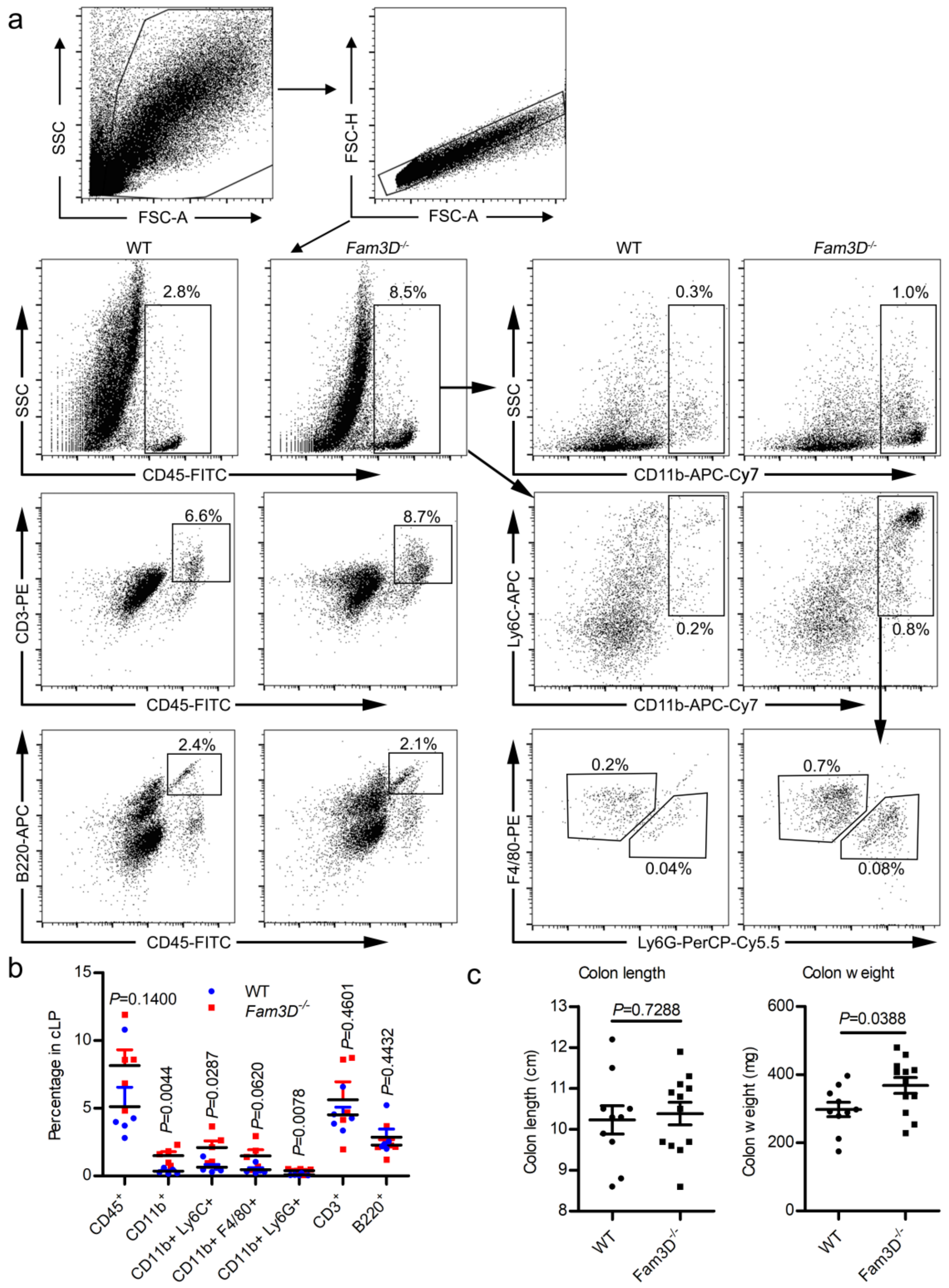

Supplementary Figure 3. Spontaneous colitis observed in aged *Fam3D*$^{-/-}$ mice

a. Representative flow cytometric analysis of colonic leukocytes infiltrating colonic lamina propria from 1-year old mice. Leukocytes ($CD45^{+}$), myeloid cells ($CD45^{+}$ $CD11b^{+}$), neutrophils ($CD45^{+}CD11b^{+}Ly6C^{+}Ly6G^{+}F4/80^{-}$), monocytes/macrophages ($CD45^{+}CD11b^{+}Ly6C^{+}Ly6G^{-}F4/80^{+}$), T cells ($CD45^{+}CD3^{+}$) and B cells ($CD45^{+}B220^{+}$). **b.** Quantification of infiltrating colonic leukocyte subsets. $n = 5$ for each group. **c.** The weight and the length of the colons measured. WT: $n = 10$, *Fam3D*$^{-/-}$: $n = 12$. Data is presented as the mean ± SEM. Statistical significance was determined by unpaired, 2-tailed Student's *t* test.

Supplementary Figure 4

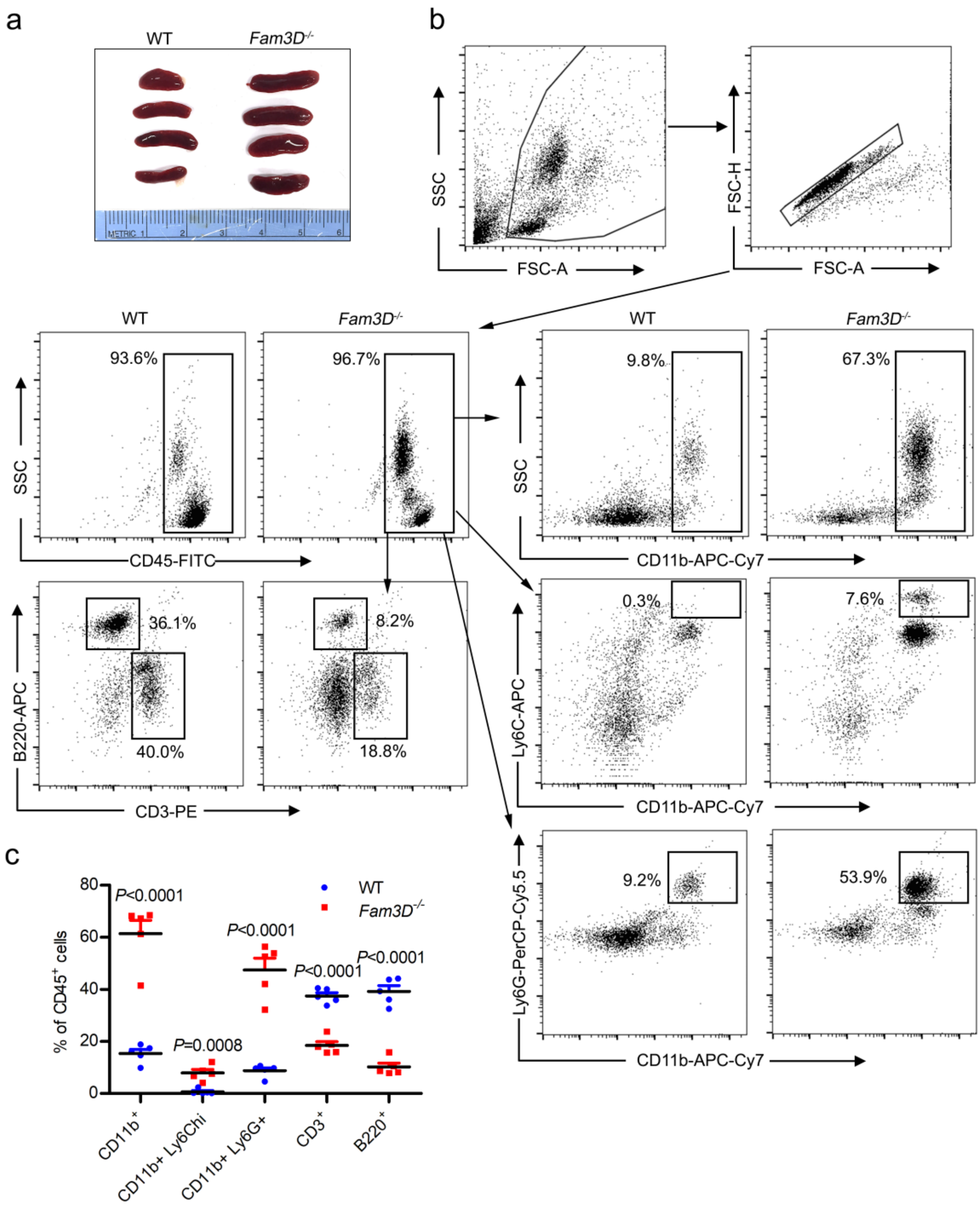

Supplementary Figure 4. Global inflammation in aged *Fam3D$^{-/-}$* mice

a. Representative spleens from aged WT and *Fam3D$^{-/-}$* mice. n = 4 for each group. **b.** Representative flow cytometric analysis of peripheral blood of aged *Fam3D$^{-/-}$* mice. Shown are: leukocytes ($CD45^+$), myeloid cells ($CD45^+$ $CD11b^+$), monocytes ($CD45^+$ $CD11b^+$ $Ly6C^{hi}$), neutrophils ($CD45^+CD11b^+Ly6G^+$), T cells ($CD45^+CD3^+$) and B cells ($CD45^+B220^+$). **c.** Quantification of different leukocyte subsets in peripheral blood. n = 5 for each group. Data is presented as the mean ± SEM. Statistical significance was determined by unpaired, 2-tailed Student's t test.

Supplementary Figure 5

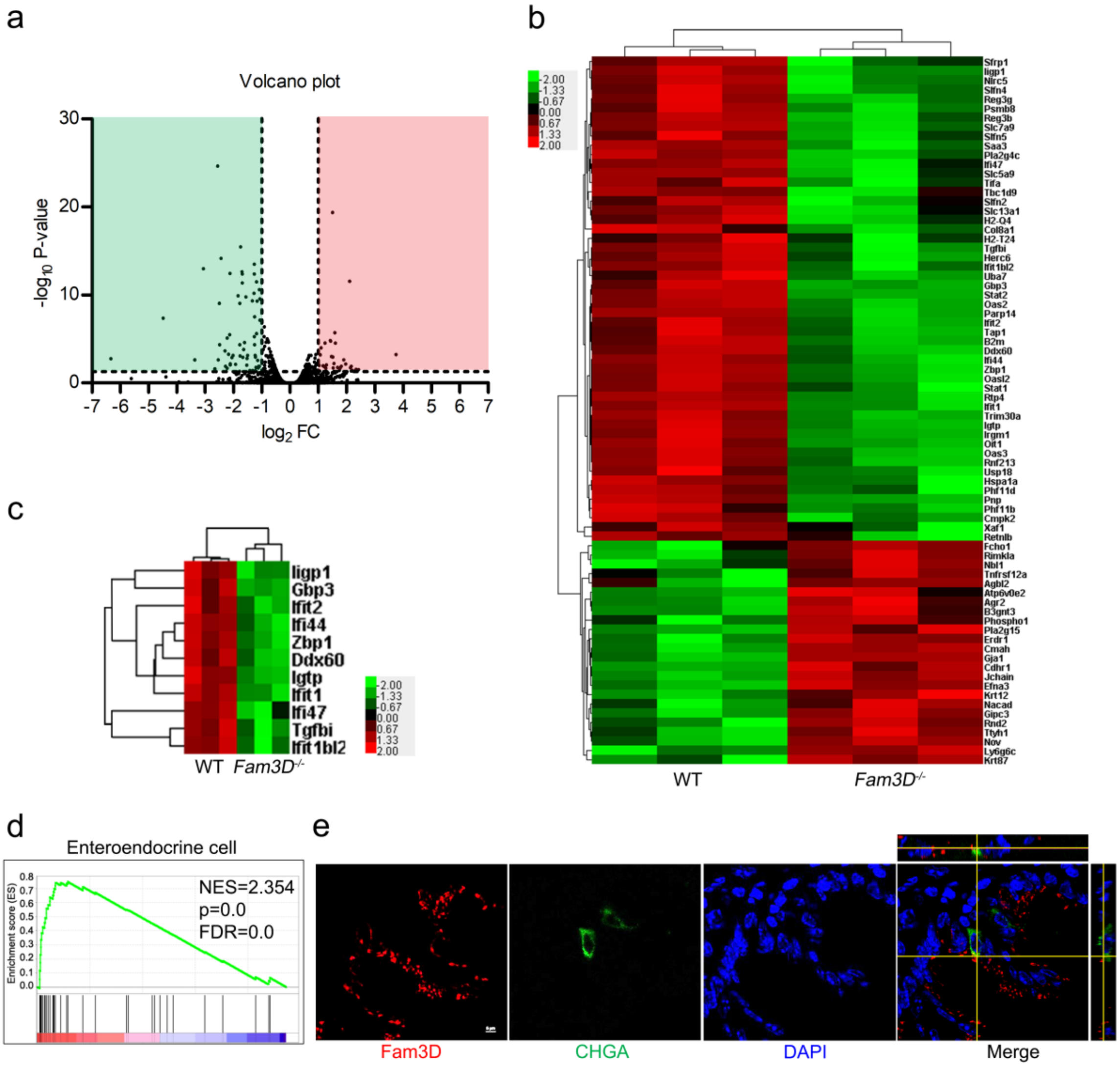

Supplementary Figure 5. Whole-transcriptome RNA-seq analysis of epithelial cells from WT and *Fam3D*$^{-/-}$ mice

a. Volcano plots showing $\log_2$ fold-change in *Fam3D*$^{-/-}$/WT expression (x axis) and $-\log_{10}$ of *P* value (*y* axis). Significantly altered transcripts (*P* adj < 0.05) with a $\log_2$ fold-change $\in$ [-1, 1]. Green area indicates down-regulated genes; red area indicates up-regulated genes in colonic epithelial cells of *Fam3D*$^{-/-}$ mice. **b.** Differentially expressed genes in colonic epithelial cells (> 1 $\log_2$ FC and < -1 $\log_2$ FC, < 0.05 adjusted *P* value). *n* = 3 for each group. **c.** Heatmaps of differentially expressed genes enriched in response to type II IFN. **d.** GSEA analysis of enteroendocrine cell signature was specifically enriched in *Fam3D*$^{-/-}$ mice. **e.** Immunofluorescent staining for Fam3D and CHGA in normal colon tissue.

Supplementary Figure 6

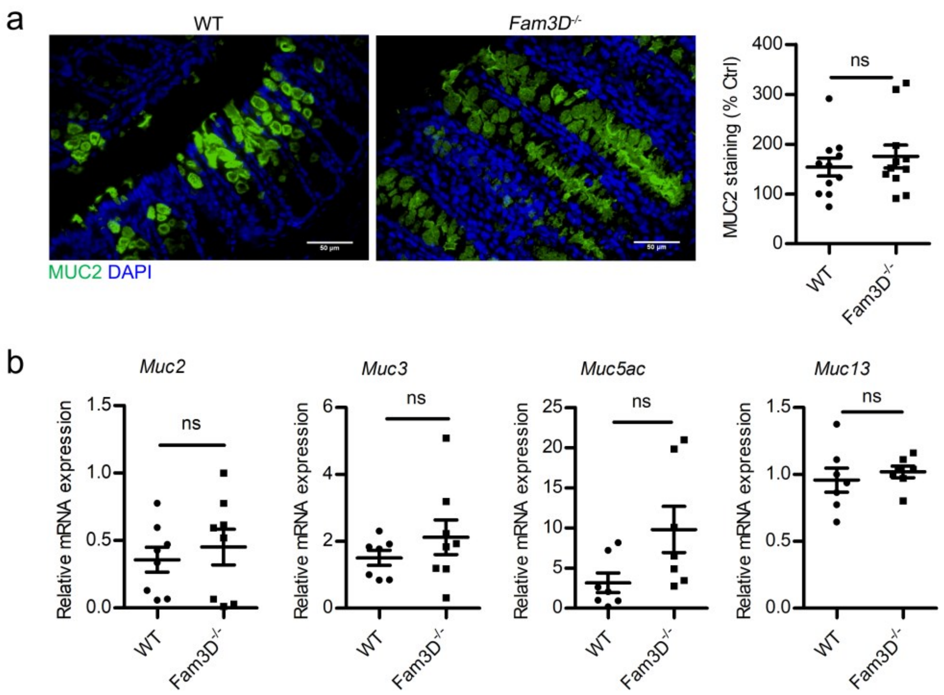

Supplementary Figure 6. Normal expression of major intestinal mucins and differentiation of secretory colonic epithelial cells in *Fam3D*$^{-/-}$ mice

a. Immunofluorescent staining for MUC2 in the colon of WT and *Fam3D*$^{-/-}$ mice. **b.** mRNA expression of genes encoding major intestinal mucins, *Muc2*, *Muc3*, *Muc5ac* and *Muc13*. n = 8 for each group. Data are representative of 1 experiment examined over 2 independent experiments. Data is presented as the mean ± SEM. Statistical significance was determined by unpaired, 2-tailed Student's t test, ns, no significance.

Supplementary Figure 7

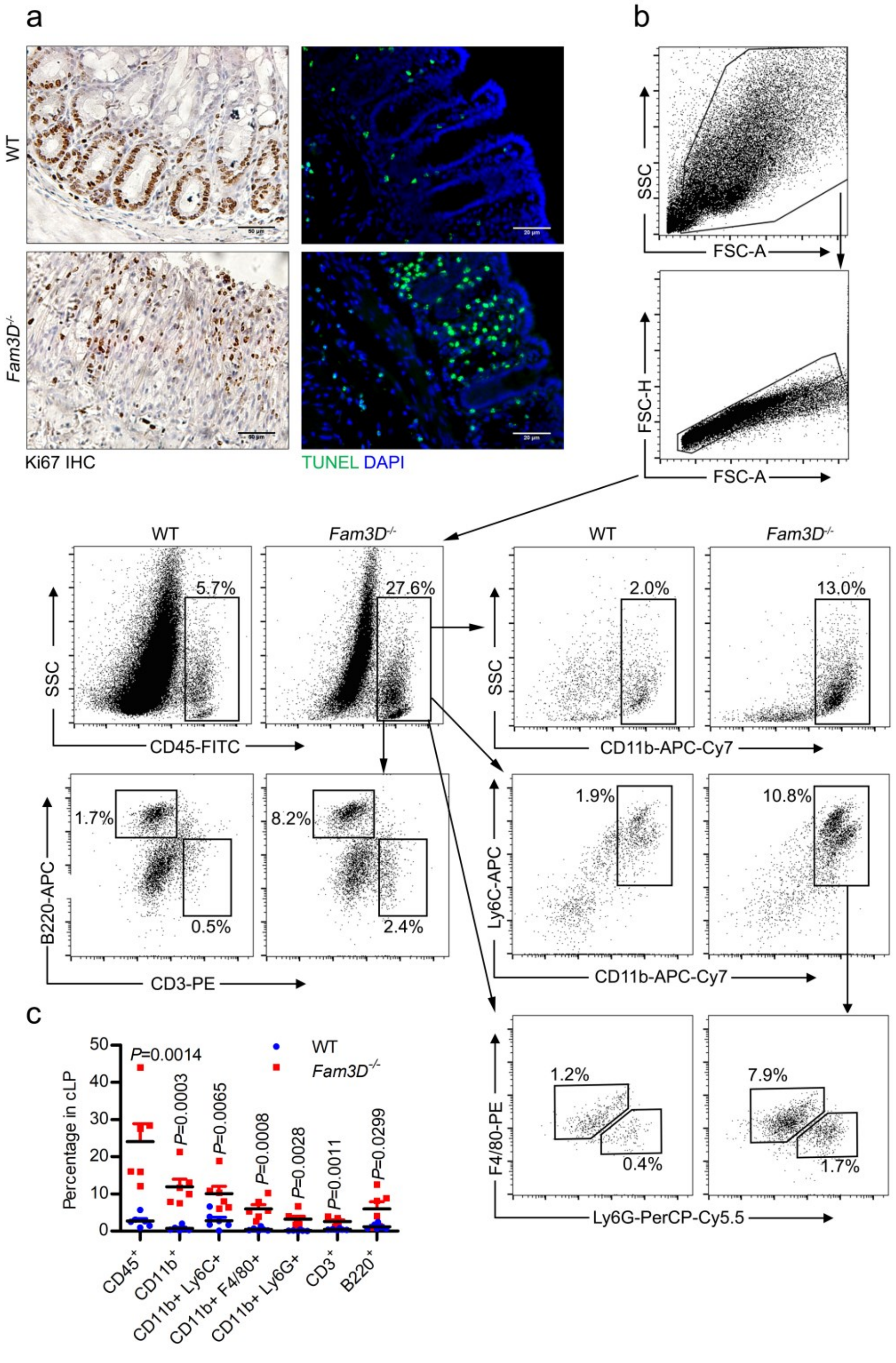

Supplementary Figure 7. Increased neutrophil recruitment into the colonic lamina propria of *Fam3D*$^{-/-}$ mice

a. Proliferation and apoptosis of colonic epithelial cells measured by Ki67 (left panel) and TUNEL staining (right panel). Scale bar = 50 μm. Data is representative of 1 experiment repeated 3 independent times. **b.** Representative flow cytometric analysis of colonic leukocytes infiltrating colonic lamina propria of mice treated with DSS at day 7. **c.** Quantification of different subsets of infiltrating leukocytes. n = 6 for each group. Data is presented as the mean ± SEM. Statistical significance was determined by unpaired, 2-tailed Student's t test.

Supplementary Figure 8

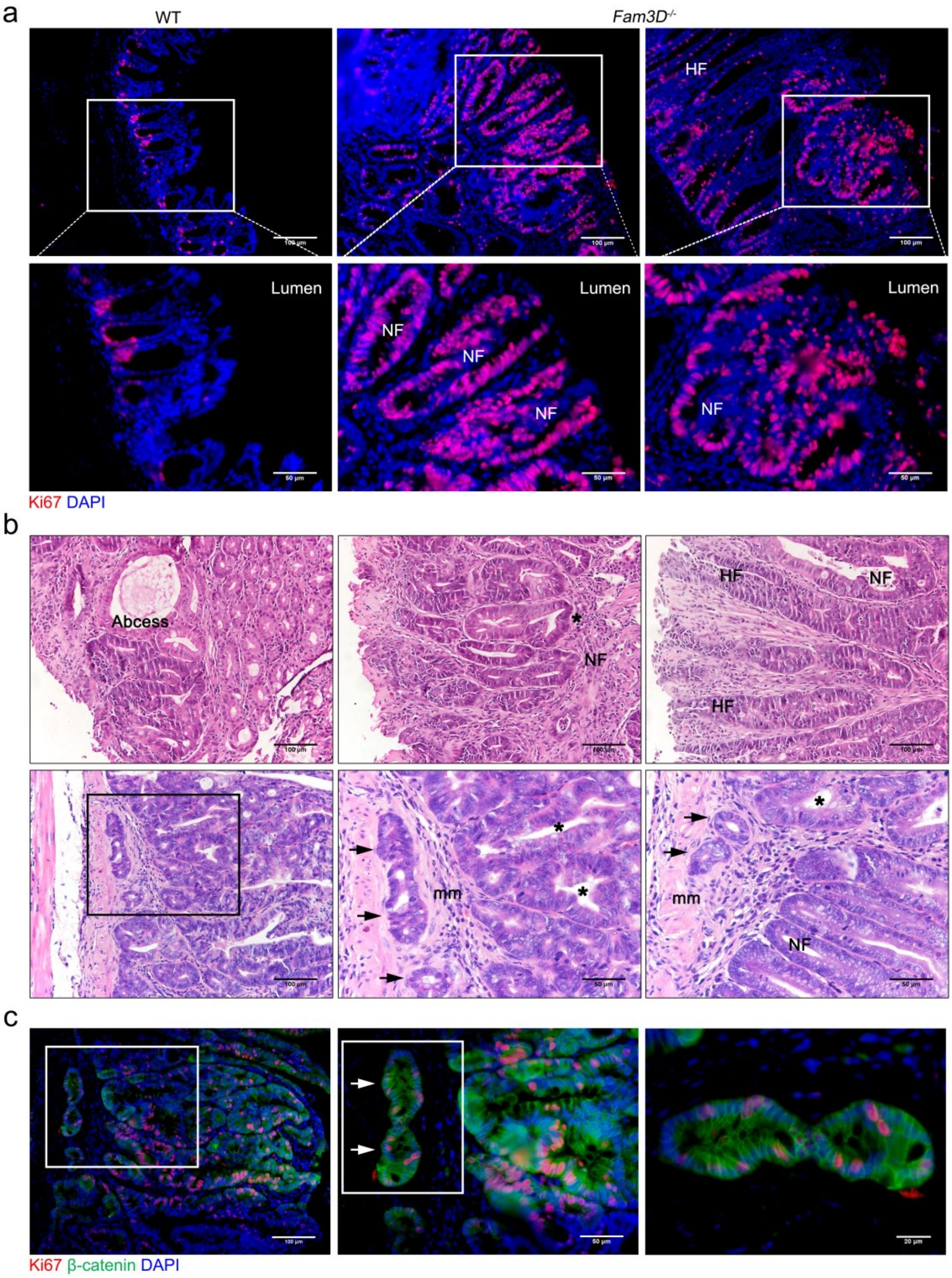

Supplementary Figure 8. Pathologic characterization of DSS-induced chronic colitis and AOM/DSS-induced tumorigenesis

a. Immunofluorescent staining of distal colonic tissue from *Fam3D*$^{-/-}$ mice with DSS-induced chronic colitis. Ki67: red; DAPI: blue. NF: neoplastic focus; HF: hyperplastic focus. Scale bar = 100 μm; Inset scale bar = 50 μm. **b.** H&E staining of distal colonic tissues of *Fam3D*$^{-/-}$ mice with AOM/DSS-induced tumors. NF: neoplastic foci; HF: hyperplastic foci; asterisk: atypical glandular architecture; mm: muscularis mucosa; arrow: submucosal gland. Scale bar = 100 μm; Inset scale bar = 50 μm. **c.** Representative images of Ki67 (red) and β-catenin (green) in the distal colon of *Fam3D*$^{-/-}$ mice with AOM/DSS-induced tumors. Submucosal gland (arrow) displaying few positive nuclei and β-catenin locating to the membrane of epithelial cells without nuclear accumulation. Scale bar = 100 μm; Inset scale bar = 50 μm (middle panel) and 25 μm (right panel). Data is representative of 1 experiment repeated 3 independent times.

Supplementary Figure 9

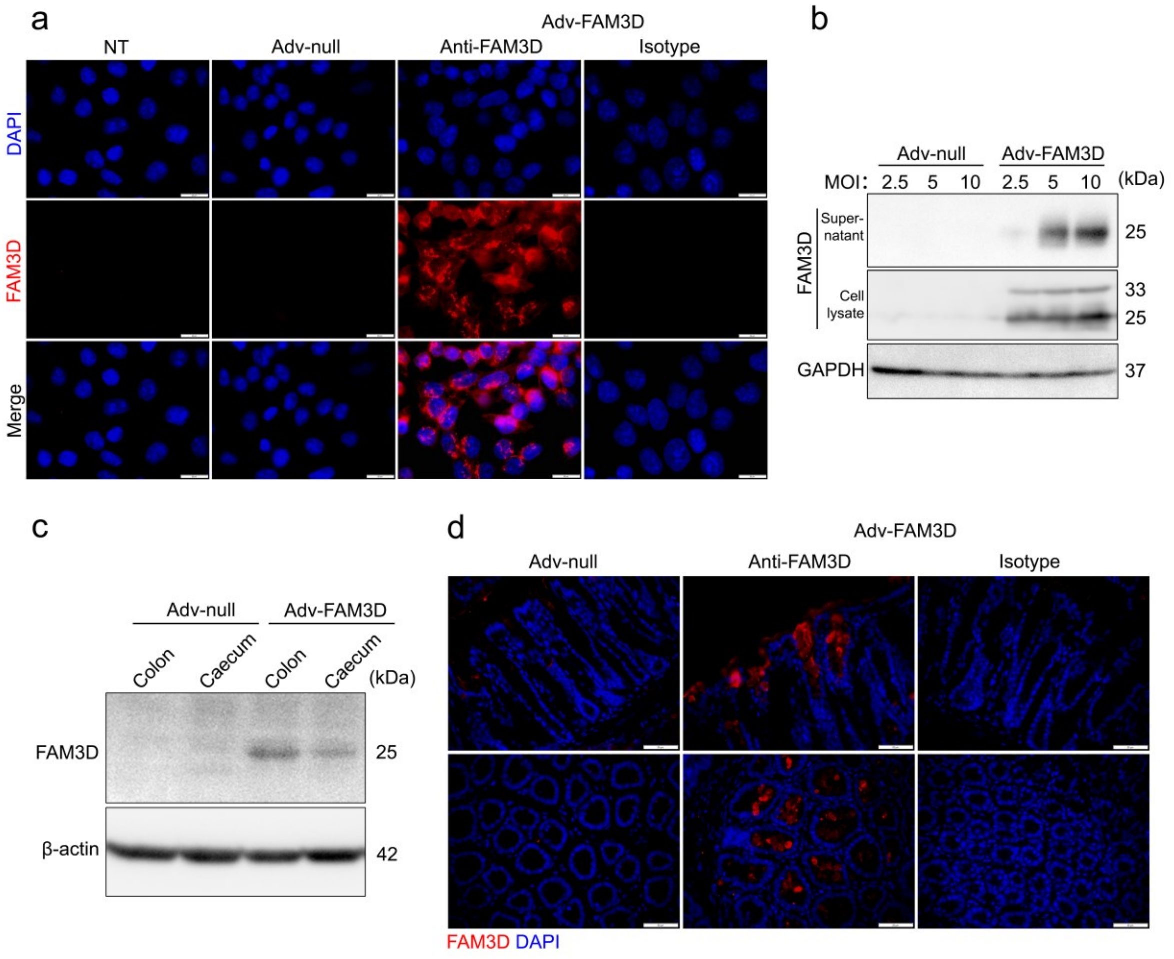

Supplementary Figure 9. Expression of adenovirus Adv-FAM3D in colon epithelial cells *in vitro* and *in vivo*

a. Immunofluorescent straining for FAM3D in a human colonic epithelial cell line FPCK-1-1 untreated (NT) or infected with adenovirus Adv-FAM3D and Adv-null control. Scale bar = 20 μm. **b.** Western blot detection of overexpressed FAM3D in the supernatant and cell lysate of FPCK-1-1 cells infected with adenovirus. **c.** Western blot of mouse colon tissues infected with Adv-FAM3D or Adv-null through intrarectal infusion. **d.** Immunofluorescent staining of FAM3D in the colon of mice infused with Adv-FAM3D or Adv-null through the rectum. Isotype: control IgG. Scale bar = 50 μm. Data is representative of 1 experiment repeated 2 independent times.

Supplementary Figure 10

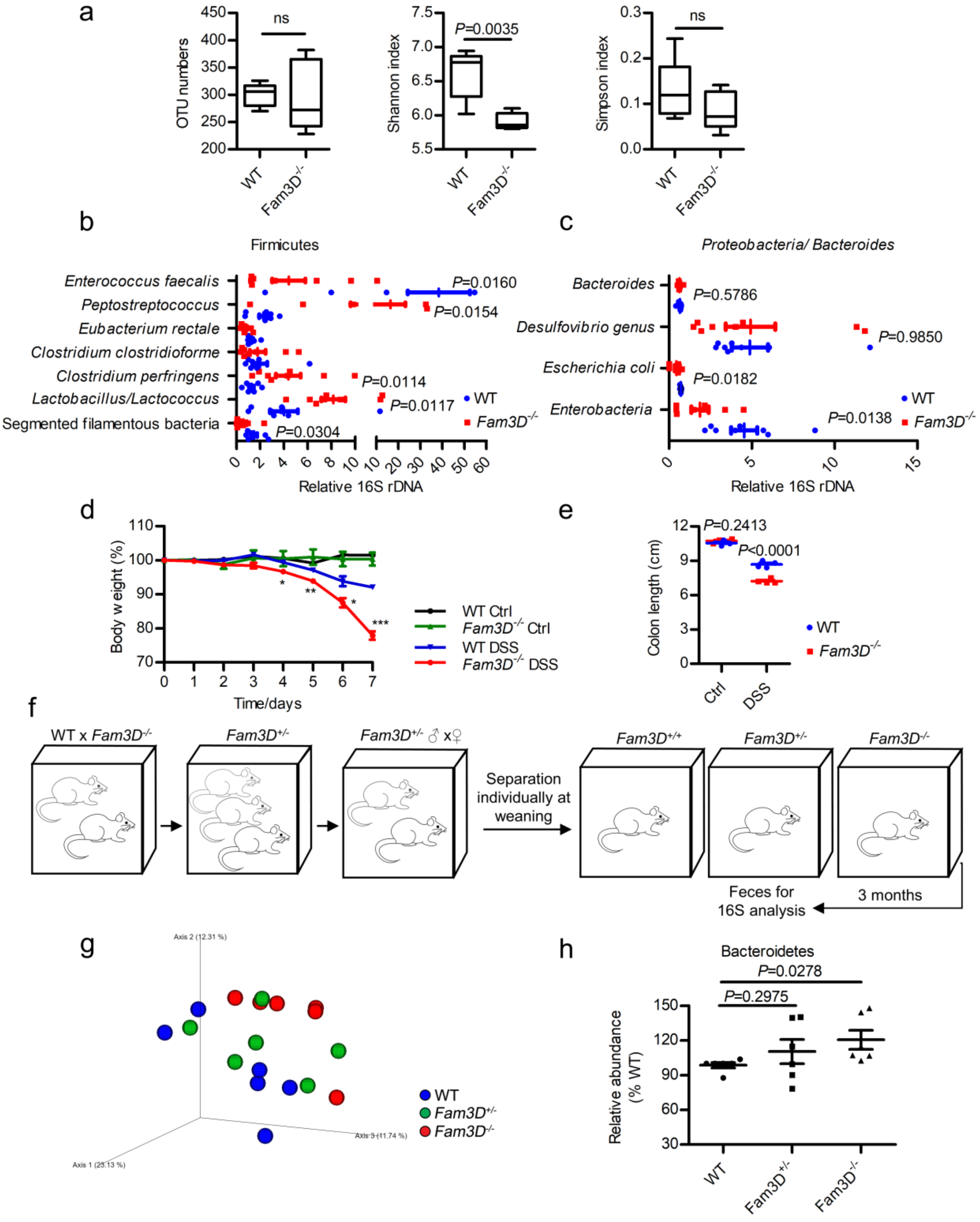

Supplementary Figure 10. Analysis of the α diversity of microbiome between WT and *Fam3D*$^{-/-}$ mice with confirmation of dysbiosis in *Fam3D*$^{-/-}$ mouse colon by real-time PCR

a. OTU numbers, Shannon diversity index and Simpson index of microbiota in WT and *Fam3D*$^{-/-}$ mouse colon. OUT: operational taxonomic unit. All box plots include the median line, the box denotes the interquartile range, whiskers denote the rest of the data distribution. **b-c.** Real-time PCR confirmation of dysbiosis in *Fam3D*$^{-/-}$ mouse colon. $n = 8$ for each group. **d-e.** Quantification of body weight and colon length of WT and *Fam3D*$^{-/-}$ mice with DSS treatment after 3-month separation. $n = 8$ for each group. **f.** Scheme for littermate single-housing. **g.** Principal-component analysis of Bray-Curtis in WT, *Fam3D*$^{+/-}$ and *Fam3D*$^{-/-}$ littermates after 3-month single-housing (6 groups). **h.** Relative abundance of Bacteroidetes in WT, *Fam3D*$^{+/-}$ and *Fam3D*$^{-/-}$ littermates after 3-month single-housing. Data is presented as the mean ± SEM. Statistical significance was determined by paired, 2-tailed Student's *t* test, , $^{*}P < 0.05$, $^{**}P < 0.01$, $^{***}P < 0.001$.

Supplementary Figure 11

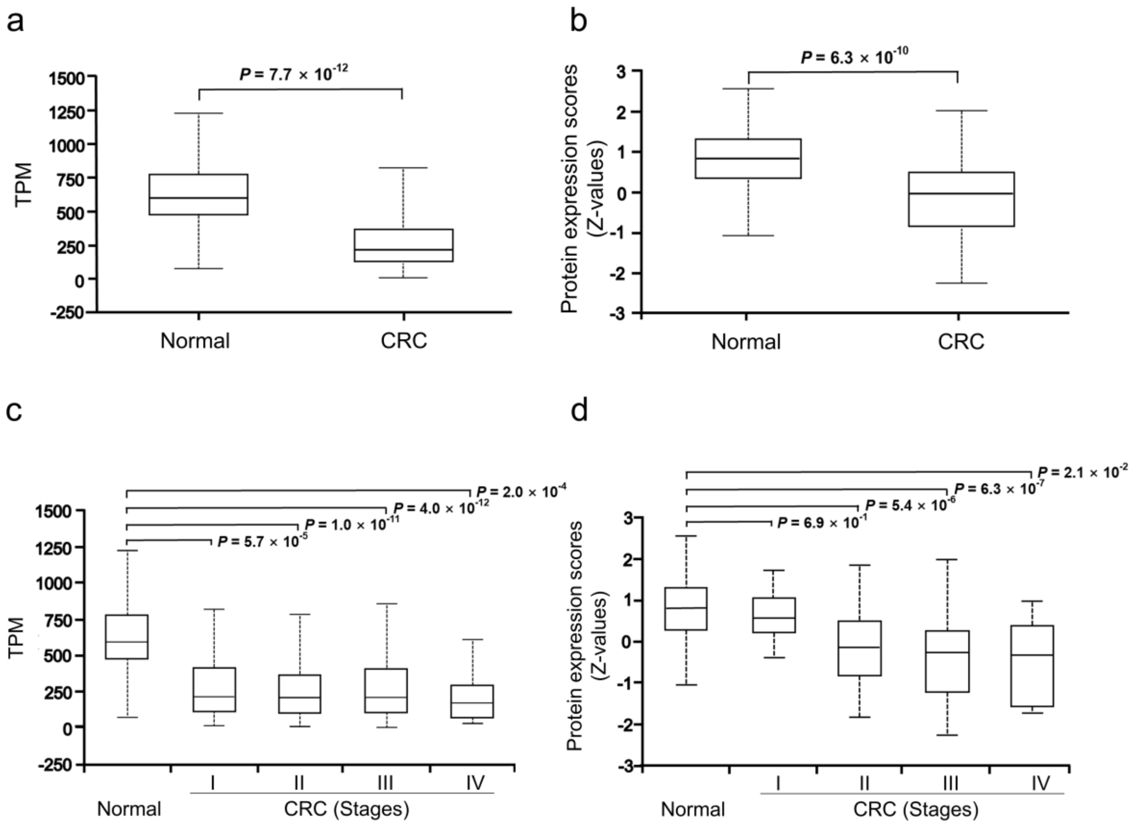

Supplementary Figure 11. Decreased expression of FAM3D transcripts and protein in human colorectal cancer (CRC)

a. TCGA RNA-sequencing results of FAM3D mRNA in human CRC (n = 286) in comparison with normal colon tissues (Normal, n = 41). **b.** FAM3D protein expression in human CRC (n = 97) in comparison with normal colon tissues (Normal) (n = 100), based on data from CPTAC Confirmatory/Discovery dataset. **c.** The expression of FAM3D mRNA in normal colon tissues (Normal) compared with CRC at different disease stages based on TCGA RNA-sequencing data (sample sizes: Normal = 41; CRC Stage I = 45, II = 110, III = 80, and IV = 39). **d.** The expression of FAM3D protein in normal colon tissues (Normal) compared with CRC at different disease stages based on CPTAC database (sample sizes: Normal = 100; CRS Stage I = 10, II = 39, III = 40, and IV = 8). Graphs were modified from *http:ualcan.path.uab.edu.*

Supplementary Figure 12

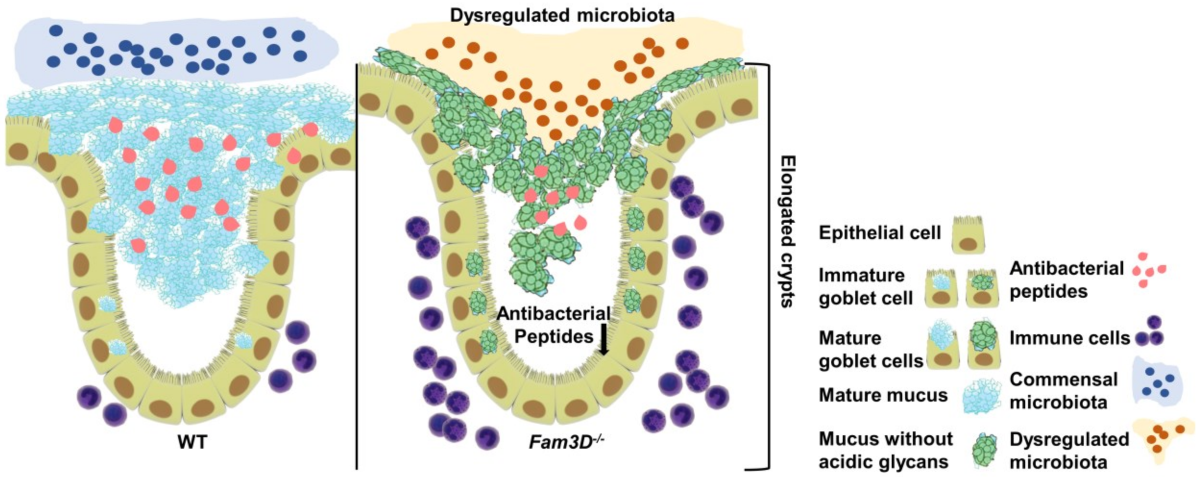

Supplementary Figure 12. Graphic abstract

FAM3D as a unique protector of the colon. Fam3D in mice is capable of sustaining the normal differentiation of goblet cells, their mucin production, and the level of anti-microbial peptides, critical for a balanced microbiome in the gut necessary for anti-inflammatory and anti-cancer host responses. In human, FAM3D is more highly expressed in normal colon with progressive reduction in CRC. Therefore, FAM3D (Fam3D) represents a new guardian identified in the gut.

Supplementary table 1. Primers used in this study

Genes	Forward primer (5'-3')	Reverse primer (5'-3')
**Quantitative PCR Primers for Mouse**		
Fam3D	GCAGTTGGCTGGGTTAAAGAC	GGATCAGACCTGCCACTCT
Muc2	CCTTCGACGGGAAGCATTAT	AATGCTGAAGGAACCAGTAGAG
Muc3	GGTGGAGAGCGTAGAGATAGA	CTTCAGACTTTCGGTCCTGTAG
Muc5ac	CTGTAACACCCAGTGTCCTAAG	AGGCTGGTAGAAGTAGGTAGAA
Muc13	GGACCCAGGCAAATGACAATA	CCCTGCTTTCCTACCAACTAAC
Reg3b	AATGGAGGTGGATGGGAATG	CCACAGAAAGCACGGTCTAA
Reg3g	CTTCCTGTCCTCCATGATCAAA	CCACCTCTGTTGGGTTCATAG
Saa1	ACACTGACATGAAGGAAGCTAAC	CCTCTGCCGAAGAATTCCTGA
Saa3	AGCCAAAGATGGGTCCAGTT	TCAGAGTAGGCTCGCCACAT
Defb1	TCATCTGTCAGCCCAACTACC	CGGAGACAGAATCCTCCATGT
Defa2	GGCTCCTGCTCACCAATTCT	GATCAGCCTGGACCTGGAAG
Defa3	TCGCTGAACATGGAGACCAC	CGAGGTAGTCATCAGGCACC
Defa21	CGCTGAGAGTGCAGATGACA	GAAGTGTTCATCAGGCCCCA
Defa24	ACACTGAGCTGCTACTCACC	AGACACAGCCTGGTCCTCTT
Cramp	CTTCAAGGAACAGGGGGTGG	ACCTTTGCGGAGAAGTCCAG
Il1	GGGCTGGACTGTTTCTAATGC	CTTGTGACCCTGAGCGACC
Cxcl1	CACCCAAACCGAAGTCATAGC	GAAGCCAGCGTTCACCAGA
Cxcl2	GACAGAAGTCATAGCCACTCTC	GCCTTGCCTTTGTTCAGTATC
Tnfa	CTACCTTGTTGCCTCCTCTTT	GAGCAGAGGTTCAGTGATGTAG
Ccl2	TGTGCTGACCCCAAGAAGG	GGTGGTTGTGGAAAAGGTAGTG
Gapdh	ATCAAGAAGGTGGTGAAGCA	AGACAACCTGGTCCTCAGTGT
**Bacterial 16S rRNA gene primers**		
**All bacteria**	ACTCCTACGGGAGGCAGCAGT	ACTCCTACGGGAGGCAGCAGT
**Bacteroidetes**	AACGCTAGCTACAGGCTTAAC	ACGCTACTTGGCTGGTTCA
**Firmicutes**	GCGTGAGTGAAGAAGT	CTACGCTCCCTTTACAC
**Segmented filamentous bacteria (SFB)**	GACGCTGAGGCATGAGAGCA	GACGGCACGGATTGTTATTC
Lactobacillus/Lactococcus	AGCAGTAGGGAATCTTCCA	CACCGCTACACATGGAG
Eubacterium rectale	CGGTACCTGACTAAGAAGC	CCTAGTATTCATCGTTTACGGCGTG
Enterococcus faecalis	GCTTTCGGGTGTCGCTGATG	CGTCCTTGTTCTTCTCTAAC
**Enterobacteria**	CATTGACGTTACCCGCAGAAGAAGC	CTCTACGAGACTCAAGCTTGC
Clostridium perfringens	CGCATAACGTTGAAAGATGG	CCTTGGTAGGCCGTTACCC
Clostridium clostridioforme	AATCTTGATTGACTGAGTGGCGGAC	CCATCTCACACTACCGGAGTTTTTC
Faecalibacterium prausnitzii	GATGGCCTCGCGTCCGATTAG	CCGAAGACCTTCTTCCTCC
**Bacteroides**	GTCAGTTGTGAAAGTTTGC	CAATCGGAGTTCTTCGTG
Desulfovibrio genus	CCGTAGATATCTGGAGGAACATCAG	CCGTAGATATCTGGAGGAACATCAG
Peptostreptococcus anaerobius	GCTCGGTGCCTTCACTAACG	AGCCCCGAAGGGAAGGTGTG
E. coli	TGGCTCAGGACGAACGCTGGCGGC	CCTACTGCTGCCTCCCGTAGGAGT

Supplementary table 2. Disease score indexes

Score	Weight loss	Stool consistency	Blood
0	None	Normal	Negative hemocult
1	1–5%	Soft but still formed	Negative hemocult
2	6–10%	Soft	Positive hemocult
3	11-18%	Very soft; wet	Blood traces in stool visible
4	>18%	Watery diarrhea	Gross rectal bleeding
