# Supplementary material for: FAM3D is essential for colon homeostasis and host defense against inflammation associated carcinogenesis
Source: Nat Commun. 2020 Nov 20;11:5912. doi: 10.1038/s41467-020-19691-z (PMC7679402; doi:10.1038/s41467-020-19691-z)
Supplement: Supplementary file 3 — Reporting Summary [file 41467_2020_19691_MOESM3_ESM.pdf]

nature research

Corresponding author(s): Ji Ming Wang; Ying Wang

Last updated by author(s): Aug 22, 2020

Reporting Summary

Nature Research wishes to improve the reproducibility of the work that we publish. This form provides structure for consistency and transparency in reporting. For further information on Nature Research policies, see our Editorial Policies and the Editorial Policy Checklist.

Statistics

For all statistical analyses, confirm that the following items are present in the figure legend, table legend, main text, or Methods section.

n/a	Confirmed	
☐	☒	The exact sample size (*n*) for each experimental group/condition, given as a discrete number and unit of measurement
☐	☒	A statement on whether measurements were taken from distinct samples or whether the same sample was measured repeatedly
☐	☒	The statistical test(s) used AND whether they are one- or two-sided<br>*Only common tests should be described solely by name; describe more complex techniques in the Methods section.*
☐	☒	A description of all covariates tested
☐	☒	A description of any assumptions or corrections, such as tests of normality and adjustment for multiple comparisons
☐	☒	A full description of the statistical parameters including central tendency (e.g. means) or other basic estimates (e.g. regression coefficient) AND variation (e.g. standard deviation) or associated estimates of uncertainty (e.g. confidence intervals)
☐	☒	For null hypothesis testing, the test statistic (e.g. *F*, *t*, *r*) with confidence intervals, effect sizes, degrees of freedom and *P* value noted<br>*Give P values as exact values whenever suitable.*
☐	☒	For Bayesian analysis, information on the choice of priors and Markov chain Monte Carlo settings
☐	☒	For hierarchical and complex designs, identification of the appropriate level for tests and full reporting of outcomes
☐	☒	Estimates of effect sizes (e.g. Cohen's *d*, Pearson's *r*), indicating how they were calculated

Our web collection on statistics for biologists contains articles on many of the points above.

Software and code

Policy information about availability of computer code

Data collection	Western blotting images were collected by G:Box gel doc system (Syngene, Frederick, MD); qPCR data were collected by 7500 Real-Time PCR system (Applied Biosystems, NY); Color stained samples for histological evaluation and fluorescence samples for molecular based evaluation were imaged by using Olympus DP80 (Japan); Confocal images were collected by using immunofluorescence confocal microscopy Leica SP5 (Leica Microsystems Inc, IL); Flow cytometry data were collected by using FACSVerseTM System (BD, NJ); In RNA-seq analysis, reads were acquired on an Illumina HiSeq 2000 (San Francisco, CA); In microbiome-seq analysis, reads were acquired on an Illumina MiSeq platform (San Francisco, CA).
Data analysis	Western blotting results were analyzed by using ImageJ (NIH, MD); qPCR data was analysis by using Excel (Microsoft, Seattle, WA); Histological and fluorescence images were analyzed by using ImageJ (NIH, MD). Flow cytometry data were analyzed by using FlowJo (X10.0.7; BD, NJ); In RNA-seq analysis, acquired reads were aligned to the GRCm38 mouse genome assembly using TopHat2 and counted using htseq. Differential expression gene analysis was performed using edgeR (v3.6.3). Data were plotted using R. Gene set enrichment analysis (GSEA) was performed (http://www.broadinstitute.org/gsea/index.jsp); In microbiome-seq analysis, fastq files were pre-processed and analyzed using QIIME2 version 2-2018.2 (https://qiime2.org). The DADA2 algorithm implemented in QIIME2. Taxonomic classification was performed using the QIIME2 feature-classifier (https://github.com/qiime2/q2-feature-classifier) plugin trained on the Silva 132 database. Transcripts Per Million values were compared to estimate relative abundance of the transcripts for cells that have approximately the same number of transcripts per-cell (http:ualcan.path.uab.edu). The significance of the difference in FAM3D expression at mRNA levels between normal tissues and CRC samples as well as the subgroups was assessed by t test using a PERL script with Comprehensive Perl Archive Network module "Statistics:: TTest". Statistical analysis was performed by using GraphPad Prism V5.0 (San Jose, CA).

For manuscripts utilizing custom algorithms or software that are central to the research but not yet described in published literature, software must be made available to editors and reviewers. We strongly encourage code deposition in a community repository (e.g. GitHub). See the Nature Research guidelines for submitting code & software for further information.

Data

Policy information about availability of data

All manuscripts must include a data availability statement. This statement should provide the following information, where applicable:

- Accession codes, unique identifiers, or web links for publicly available datasets
- A list of figures that have associated raw data
- A description of any restrictions on data availability

The authors declare that the most data supporting the findings of this study are available within the paper and its supplementary information files. Signature genes that each type of intestinal epithelial cells, which were used for GSEA analysis, were reported in Yan et al., 2017 (doi: 10.1038/nature22313). The expression of FAM3D transcripts and protein was analyzed using data obtained from the Cancer Genome Atlas (TCGA) and Clinical Proteomic Tumor Analysis Consortium (CPTAC) Confirmatory/Discovery dataset.

Field-specific reporting

Please select the one below that is the best fit for your research. If you are not sure, read the appropriate sections before making your selection.

- [x] Life sciences
- [ ] Behavioural & social sciences
- [ ] Ecological, evolutionary & environmental sciences

For a reference copy of the document with all sections, see nature.com/documents/nr-reporting-summary-flat.pdf

Life sciences study design

All studies must disclose on these points even when the disclosure is negative.

Sample size	We usually used at least 3 mice/group to ensure the statistically significant difference could be obtained from two-tailed Student's t-test. However, we also tried to minimize the animal number to conform to the guidelines for the animal experiments. Exact numbers of animals used in individual experiments are indicated in figure legends.
Data exclusions	No data were excluded from the analyses.
Replication	All attempts at replication were successful. Exact replications are indicated in figure legends.
Randomization	Usually, we randomly chose mice from the same or different littermates for each experiment group and also randomly chose the control mice with same sex and similar date of birth.
Blinding	The investigators were blinded to group allocation during data collection and analysis.

Reporting for specific materials, systems and methods

We require information from authors about some types of materials, experimental systems and methods used in many studies. Here, indicate whether each material, system or method listed is relevant to your study. If you are not sure if a list item applies to your research, read the appropriate section before selecting a response.

Materials & experimental systems

n/a	Involved in the study	
[ ]	[x]	Antibodies
[ ]	[x]	Eukaryotic cell lines
[x]	[ ]	Palaeontology and archaeology
[ ]	[x]	Animals and other organisms
[x]	[ ]	Human research participants
[x]	[ ]	Clinical data
[x]	[ ]	Dual use research of concern

Methods

n/a	Involved in the study	
[x]	[ ]	ChIP-seq
[ ]	[x]	Flow cytometry
[x]	[ ]	MRI-based neuroimaging

Antibodies

Antibodies used	Goat anti-mouse FAM3D antibody (AF3027) and goat anti-human FAM3D antibody (AF2869) were purchased from R&D Systems (Minneapolis, MN). Rabbit anti-β-actin (4970) and GAPDH (5174) were purchased from Cell Signaling Technology (Boston, MA). Rat anti-mouse CD3, rabbit anti-B220, rabbit anti-F4/80, Rat anti-mouse Ly6G, rabbit anti-Ki67, rabbit anti-CHGA, goat-anti rabbit IgG horseradish peroxidase (HRP)-conjugated (ab6721) and rabbit anti-goat IgG HRP-conjugated (ab6741) were purchased from Abcam (Cambridge, UK). Rat anti-EpCAM antibody (sc-53532), mouse anti-β-catenin antibody (sc-7963) and rabbit anti-mouse Mucin 2 (MUC2) antibody were purchased from Santa Cruz (Dallas, Texas). Rabbit anti-mouse Reg3γ, goat anti-rabbit IgG Alexa Fluor 488 and 568, donkey anti-rat IgG Alexa Fluor 488 and 568, rabbit anti-goat IgG Alexa Fluor 568 antibodies were purchased from Invitrogen (San Diego, CA). Fluorescence-labelled anti-mouse CD45-FITC (103108), anti-mouse CD11b-APC-Cy7 (101226), anti-mouse F4/80-PE

	(123109), anti-mouse Ly6G-PerCP-Cy5.5 (127616),anti-mouse Ly6C-APC (128016), anti-mouse CD3-PE (100206), anti-mouse B220-APC (103212) antibodies were obtained from BioLegend (San Diego, CA).
Validation	All of antibodies used in this study can be validated on the manufacturer's website. Goat anti-mouse FAM3D antibody (AF3027) and goat anti-human FAM3D antibody (AF2869) were purchased from R&D Systems (Minneapolis, MN) were validated for IF, IHC and WB on the website and by indicated isotype controls in our systems. Rabbit anti-β-actin (4970) and GAPDH (5174) were validated on the website of Cell Signaling Technology. Rat anti-mouse CD3, rabbit anti-B220, rabbit anti-F4/80, Rat anti-mouse Ly6G, rabbit anti-Ki67, rabbit anti-CHGA, goat-anti rabbit IgG horseradish peroxidase (HRP)-conjugated (ab6721) and rabbit anti-goat IgG HRP-conjugated (ab6741) were validated for IF or IHC on the website of Abcam and also validated by indicated isotype controls in our systems. Rat anti-EpCAM antibody (sc-53532) and mouse anti-β-catenin antibody (sc-7963) and rabbit anti-mouse Mucin 2 (MUC2) antibody were validated for IF on the website of Santa Cruz, references and also validated by indicated isotype controls. Rabbit anti-mouse Reg3γ, goat anti-rabbit IgG Alexa Fluor 488 and 568, donkey anti-rat IgG Alexa Fluor 488 and 568, rabbit anti-goat IgG Alexa Fluor 568 antibodies were validated for IF on the website of Invitrogen and references. Fluorescence-labelled anti-mouse CD45-FITC (103108), anti-mouse CD11b-APC-Cy7 (101226), anti-mouse F4/80-PE (123109), anti-mouse Ly6G-PerCP-Cy5.5,anti-mouse Ly6C-APC, anti-mouse CD3-PE (100206), anti-mouse B220-APC (103212) antibodies were validated for Flow on the website of Biolegend, references and also by indicated isotype controls.

Eukaryotic cell lines

Policy information about cell lines

Cell line source(s)	293T, was purchased from ATCC; FPCK-1-1 was a kindly gift from Dr. A. Tominaga, Kochi University, Japan.
Authentication	All cell lines used in this study were authenticated by morphology check by microscope.
Mycoplasma contamination	All cell lines tested negative for mycoplasma contamination.
Commonly misidentified lines (See ICLAC register)	None.

Animals and other organisms

Policy information about studies involving animals; ARRIVE guidelines recommended for reporting animal research

Laboratory animals	Fam3D-/- mice were generated on a C57BL/6 background. we randomly chose mice from the same or different littermates for each experiment group and also randomly chose the control mice with same sex and similar date of birth. For DSS and DSS/AOM model, 6-week mice were used; for cohousing experiments, 4-week mice were used for cohousing; for goblet cell detection at different ages, mice of newborn, 2-week, 4-week, 6-week and 6-month were used. All mice were chosen with the same sex.
Wild animals	No wild animals were used in this study.
Field-collected samples	No field-collected samples were used in this study.
Ethics oversight	The experimental protocols of this study were approved by the Ethics Committee of Peking University Health Science Center (LA2016010) and the Frederick National Laboratory for Cancer Research Animal Care and Use Committee, Frederick, MD. All experiments were performed in accordance with procedures outlined in the "Guide for Care and Use of Laboratory Animals" (National Resource Council, National Academy Press, Washington D.C).

Note that full information on the approval of the study protocol must also be provided in the manuscript.

Flow Cytometry

Plots

Confirm that:

- [x] The axis labels state the marker and fluorochrome used (e.g. CD4-FITC).
- [x] The axis scales are clearly visible. Include numbers along axes only for bottom left plot of group (a 'group' is an analysis of identical markers).
- [x] All plots are contour plots with outliers or pseudocolor plots.
- [x] A numerical value for number of cells or percentage (with statistics) is provided.

Methodology

Sample preparation	Isolation of colonic epithelial cells (CECs) and lamina propria (cLP) leukocytes was performed as previously described (DOI: 10.1038/nprot.2007.315). Briefly, colons removed of fecal contents were opened longitudinally and cut into 1 cm pieces in ice-cold PBS. The pieces of intestine in 5 ml of digestion solution (5 mmol/L EDTA and 1 mmol/L dithiothreitol (DTT) in Ca2+/Mg2+-free HBSS) were placed on an orbital shaker for 20 min at 37°C at 40g. Cell suspension obtained was passed through a 100 mm cell strainer and centrifuged to obtain CECs. Then rest colon pieces were digested with 1 mg/ml type IV collagenase and 150 U/ml DNase I. The digested cell suspension was passed over a 200 µm coarse mesh and centrifugated. The cell pellet was harvested as the cells in cLP. For analysis of leukocytes from euthanized mice, peripheral blood cells were collected through cardiac puncture, followed by elimination of red blood cells by using a lysis buffer (Tiangen Biotech Inc, Beijing, China). Cells were incubated in FACS buffer with indicated antibodies for 30 min at 4°C in the dark.
Instrument	Flow cytometry data was collected by using FACSVerseTM System (BD, NJ).
Software	Flow cytometry data was analyzed by using FlowJo (BD, NJ); Statistical analysis was performed by using GraphPad Prism V5.0 (San Jose, CA).
Cell population abundance	Colonic epithelial cells were determined by Epcam positive staining. Blood and lamina propria leukocytes were determined as several subsets by indicated markers shown in Supplemental Figure 3a, 4b and 7b.
Gating strategy	FSC/SSC and FSC-H/FSC-A gates of the starting cell population in all flow relevant experiments, gated populations were defied as "positive" or "negative".

☒ Tick this box to confirm that a figure exemplifying the gating strategy is provided in the Supplementary Information.
