# Supplementary material for: FAM3D is essential for colon homeostasis and host defense against inflammation associated carcinogenesis
Source: Nat Commun. 2020 Nov 20;11:5912. doi: 10.1038/s41467-020-19691-z (PMC7679402; doi:10.1038/s41467-020-19691-z)

Full unedited blots

A. Full unedited blots for Figure 1b, anti-Fam3D (mouse FAM3D)

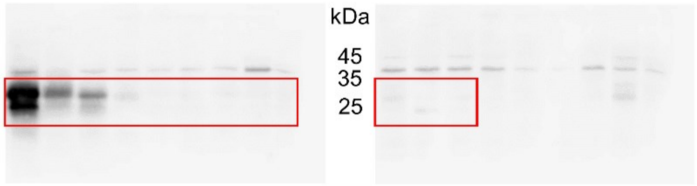

B. Full unedited blots for Figure 1b, anti-GAPDH

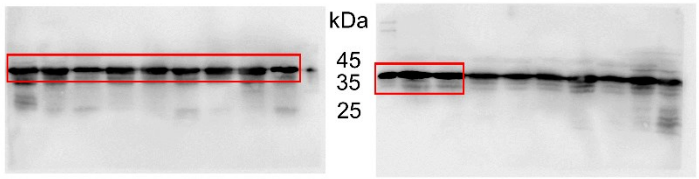

C. Full unedited blots for Figure 1e, anti-Fam3D (left) and anti-GAPDH (right)

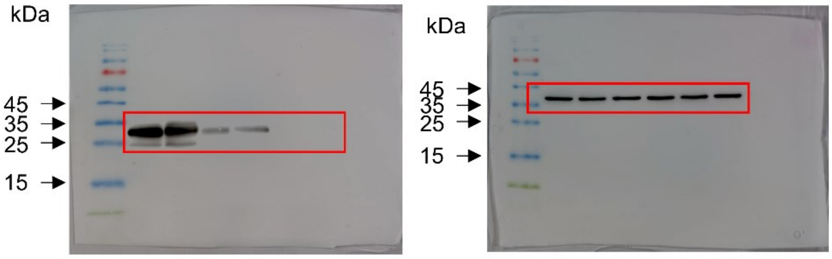

D. Full unedited blots for Supplementary Figure 1a

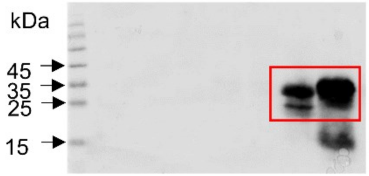

E. Full unedited blots for Supplementary Figure 1e

Anti-human FAM3D (left) and marker (right)

Anti-GAPDH (left) and marker (right)

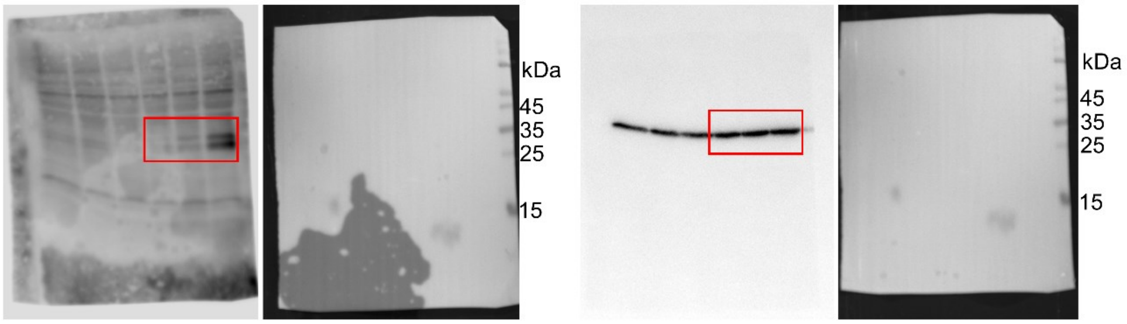

F. Full unedited blots for Supplementary Figure 9b, anti-human FAM3D in supernatant

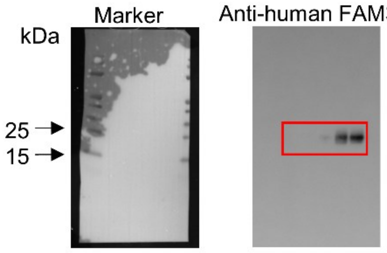

G. Full unedited blots for Supplementary Figure 9b, anti-human FAM3D in cell lysate

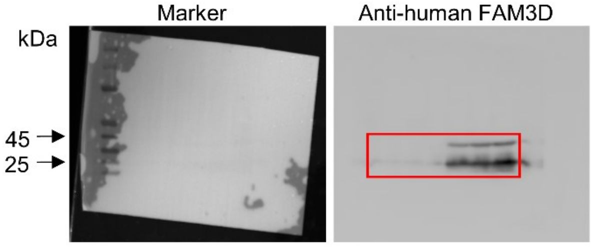

H. Full unedited blot for Supplementary Figure 9b, anti-GAPDH

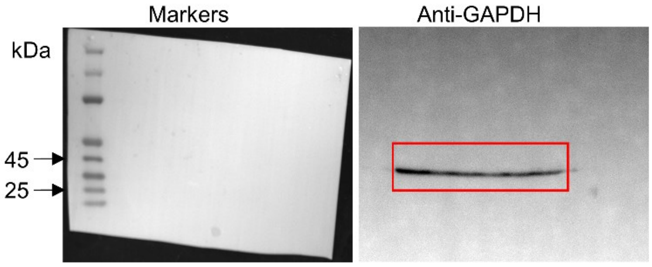

I. Full unedited blots for Supplementary Figure 9c, anti-human FAM3D in infected mouse tissues

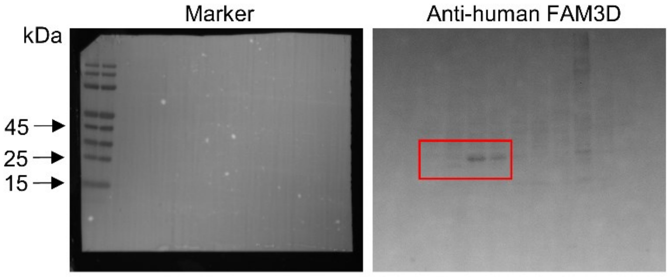

J. Full unedited blots for Supplementary Figure 9c, anti-β-actin

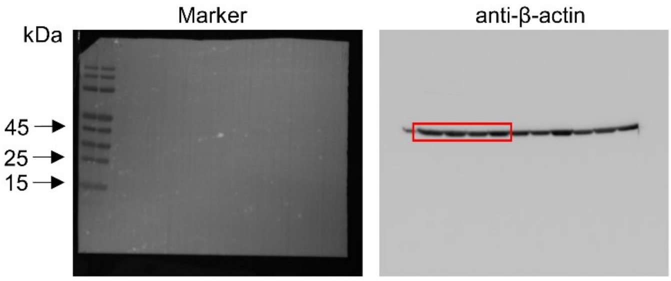

Supplement: Supplementary file 4 — Source Data [file 41467_2020_19691_MOESM4_ESM.zip › 083020 NCOMMS-19-1158189B Source Data-unedited blots.pdf]
